# The cat’s out of the bag: *Toxoplasma gondii* provides further insight into myeloid-mediated host defense

**DOI:** 10.1093/immhor/vlaf037

**Published:** 2025-08-25

**Authors:** Madison L Schanz, Fengdi Zhao, Kamryn E Zadeii, Li Chen, Américo H López-Yglesias

**Affiliations:** Department of Microbiology and Immunology, Indiana University School of Medicine, Indianapolis, IN, United States; Department of Biostatistics, University of Florida, Tallahassee, FL, United States; Department of Microbiology and Immunology, Indiana University School of Medicine, Indianapolis, IN, United States; Department of Biostatistics, University of Florida, Tallahassee, FL, United States; Department of Microbiology and Immunology, Indiana University School of Medicine, Indianapolis, IN, United States

**Keywords:** immunoparasitology, innate immunity, myeloid cells, T-bet

## Abstract

The obligate intracellular protozoan pathogen *Toxoplasma gondii* is estimated to infect a third of the world’s population. Toxoplasmosis is considered a significant worldwide disease that can lead to morbidity or death in immunocompromised individuals. Host defense against *T. gondii* has been demonstrated to be dependent on a rapid myeloid cell and lymphocyte response working in concert to quickly eliminate the invading pathogen. Classically, T-bet–dependent group 1 innate lymphocytes (ILC1s), natural killer (NK) cells, and CD4^+^ T cell–derived interferon-γ (IFN-γ) are considered indispensable for host resistance against *T*. *gondii*. However, recent discoveries have illustrated that T-bet is not required for NK cell– or CD4^+^ T cell–derived IFN-γ. Yet, lack of T-bet still results in rapid mortality, pointing to a T-bet–dependent myeloid cell–mediated host defense pathway. This review summarizes the myeloid cell–mediated immune response against *T*. *gondii* and provides insights into the lesser known components of the T-bet–dependent myeloid cell–dependent host defense pathway for pathogen clearance.

## Introduction

The apicomplexan obligate intracellular parasite and the causative agent of toxoplasmosis, *Toxoplasma gondii*, can infect virtually all nucleated cells of warm-blooded animals, including humans. *T*. *gondii* transmission typically occurs from unintentional ingestion of feline fecal matter, foodborne transmission via undercooked meats or contaminated produce, and congenital transmission via infection during pregnancy that can lead to the parasite crossing the placental barrier and infecting the fetus. Approximately a third of the human population tests seropositive for the parasite, making *T*. *gondii* a global health burden and concern.

Following the consumption of the parasite, *T*. *gondii* will ultimately evade clearance and transform into slow-growing cysts within neurons and other tissues, leading to a lifelong infection.^1^ Notably, individuals that are chronically infected and develop an immunocompromised status due to medications (i.e. chemotherapy or radiation therapy), infection (i.e. HIV/AIDS), or cancer (i.e. leukemia, lymphoma, or multiple myeloma) will lead to reactivation of the parasite and uncontrolled parasite growth. Parasite reactivation can lead to toxoplasmosis encephalitis, resulting in brain inflammation and necrosis.

In immunocompetent individuals, *T*. *gondii* generally causes mild flu-like symptoms that typically go unnoticed and untreated. Mild symptoms caused by *T. gondii* are due to a rapid and robust type 1 immune response characterized by indispensable CD4^+^ T helper 1 (Th1) cell–derived interferon-γ (IFN-γ). The parasite-mediated Th1 response has been classically considered to be T-bet dependent, yet recent studies by our group and others have revealed that T-bet–independent natural killer (NK) cells, CD8^+^ T cells, and Th1 cells remain functional mediators of IFN-γ during acute infection.^2^^,^^3^ Despite the presence of NK-, CD8^+^-, and Th1-derived IFN-γ in the absence of T-bet, *Tbx21*-deficient mice rapidly succumb to *T. gondii* infection,^3–5^ suggesting a T-bet–dependent myeloid cell–mediated host defense pathway. This review examines the individual roles of myeloid cells in the innate immune response to *T*. *gondii* and aims to delineate how the transcription factor T-bet mediates myeloid cell–dependent host resistance against intracellular pathogens.

### Myeloid cell–mediated host defense

**Figure 1.**
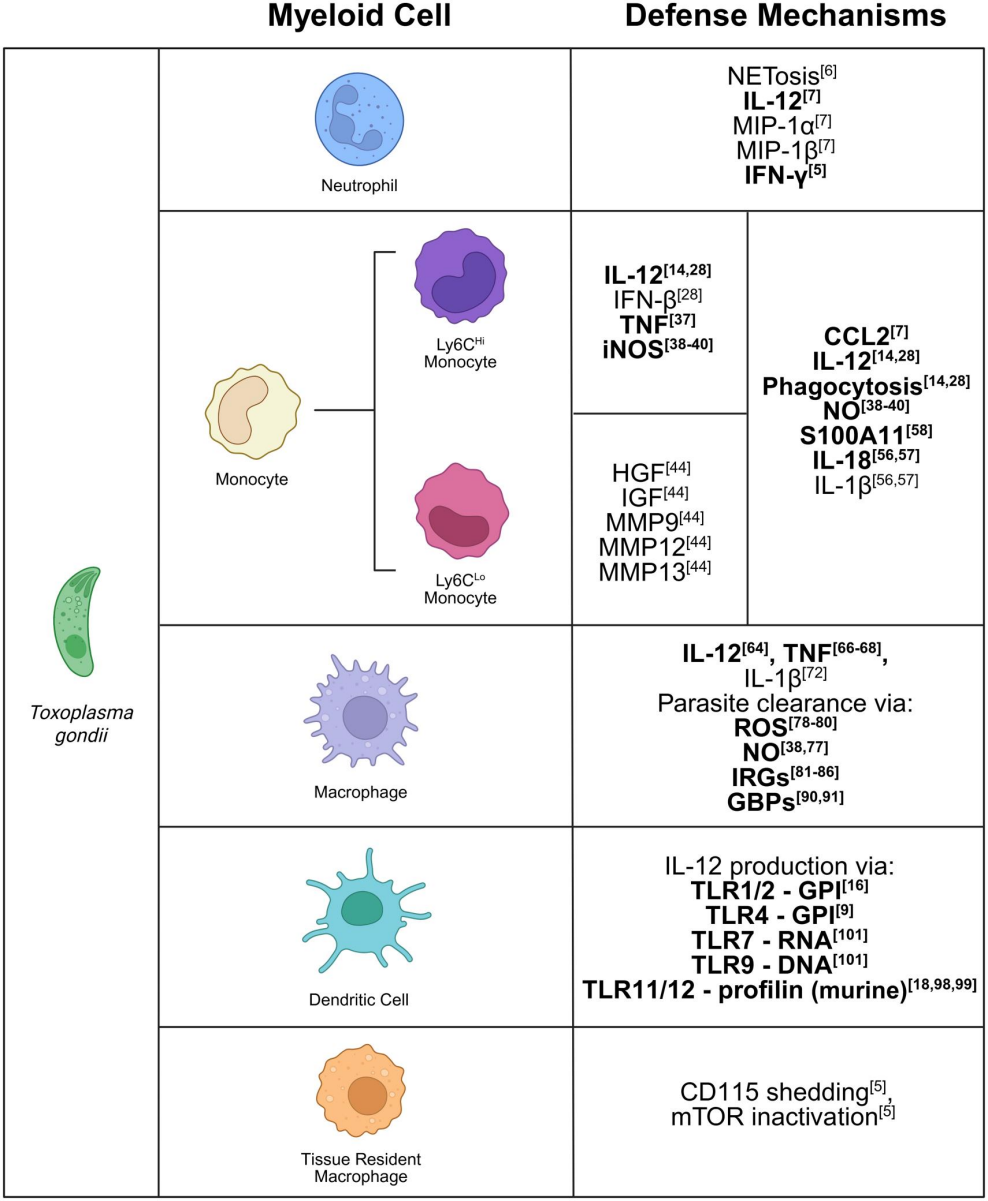
Myeloid cells involved in innate defense against *T. gondii*. Myeloid cells, such as neutrophils, monocytes, macrophages, DCs, and TRMs, employ a variety of defense mechanisms via cytokine production, chemoattractant release, and specialized antimicrobial mechanisms such as NETs, oxygen radicals, IRGs, and GBPs. This multifaceted functionality is critical for recruiting additional innate immune cells, initiating trained immunity, priming of the adaptive immune response, and ultimately, rapid parasite clearance. While the protective measures for each cell type vary greatly, overlap in overall function of such measures between multiple cell types highlights the need for redundant processes to ensure host survival. Emboldened text indicates pathways that have been established as directly relevant to host defense against *T. gondii.* Created in BioRender (https://biorender.com/c91w081).

The innate immune system is comprised of specialized myeloid cells and lymphocytes, which work in concert to rapidly clear invading pathogens. *T. gondii* is a robust inducer of the type 1 immune response, defined by IFN-γ production, which is indispensable for host resistance during infection.^6^ Our group and others have shown that during the acute stage of *T. gondii* infection, myeloid cells must work in tandem with group 1 innate lymphoid cells (ILC1s), NK cells, and Th1 cells to generate a rapid and protective IFN-γ response. During *T*. *gondii* infection, the myeloid cells that have been shown to play key roles in host defense are neutrophils, monocytes, macrophages, dendritic cells (DCs), and a recently identified subpopulation CD11c^+^MHCII^−^ myeloid cells (Fig. 1).^4^ The transcription factor T-bet, encoded by *Tbx21*, has classically been defined as the master regulator of IFN-γ; however, our group recently showed that T-bet–expressing CD11c^+^MHCII^−^ myeloid cells (TMCs) play an important role in host resistance during acute *T. gondii* infection.^4^ Determining T-bet’s role in myeloid cell–mediated innate defense is critical to further our understanding of the mechanisms that eliminate *T. gondii* and prevent the parasite’s progression to the host’s central nervous system (CNS), where it will ultimately establish a lifelong infection. Herein, we discuss the individual myeloid cells involved in mediating protective immunity against *T. gondii* to better our understanding of host defense against *T. gondii* and similar intracellular pathogens.

## Neutrophils

Neutrophils have been well-studied in host defense against numerous microbial pathogens and are critical first responders to infection. They have been proven to provide several unique antiparasitic mechanisms contributing to host defense against *T*. *gondii*. A key antimicrobial function of neutrophils is the release of neutrophil extracellular traps (NETs), weblike structures composed of DNA fibers, histones, and antimicrobial proteins that have been demonstrated to play a role in trapping viruses, bacteria, fungi, and protozoans.^7^ To elucidate the role of this specialized antimicrobial mechanism, in vitro examination of fully functional neutrophils acting against tachyzoites was compared with neutrophils with DNase-deactivated NETs.^7^ This study illustrated that NETs provide an important defensive mechanism against *T. gondii* via a mitogen-activated protein kinase and extracellular signal-regulated kinase, independent of the MyD88 pathway.^7^ These data suggest that NETs are able to kill the parasite due to its entrapment and subsequent inability to enter host cells, leaving *T. gondii* unable to harvest nutrients and proliferate.^7^ Although this mechanism provides a hypothesis for parasite elimination, the specific mechanism for NET-mediated parasite death remains unknown, and further studies are needed to elucidate the significance of NETs during *T. gondii* infection in vivo.

Additional studies have shown that neutrophils are a notable source of interleukin (IL)-12, tumor necrosis factor (TNF), MIP-1α (macrophage inflammatory protein 1α) (also known as CCL3), and MIP-1β (also known as CCL4), which are expressed independent of pathogen-associated molecular pattern (PAMP) recognition via Toll-like receptors (TLRs).^8^ These proinflammatory cytokines were demonstrated to play a key role in the recruitment of macrophages to the site of infection and in T cell activation.^9^ Moreover, murine neutrophils are capable of recognizing *T. gondii* surface GPI (glycosylphosphatidylinositol) anchors via TLR2, generating the production of CCL2, which is critical for monocyte recruitment.^8^ However, TLR2-deficient mice remain capable of initiating a protective immune response against *T. gondii*, signifying that TLR2 plays a limited role for host defense against the parasite.^10^

Early studies utilizing *Cxcr2*-deficient mice set out to investigate the role of neutrophils in host defense against an acute intraperitoneal (i.p.) *T. gondii* infection. The absence of CXCR2 resulted in defective neutrophil recruitment and an increase in parasite burden at the site of infection.^11^ Yet, CXCR2 is also expressed on basophils, monocytes, macrophages, NK, NKT, and T cell subsets. This suggests conclusions from studies utilizing *Cxcr2*^−/−^ mice could overexaggerate the role of neutrophils in host defense against *T. gondii*. Thus, to clarify the importance of neutrophils in host resistance against *T. gondii*, experimentation involving the depletion of neutrophils was necessary. By performing antibody mediated depletion using RB6-8C5 (GR-1), studies concluded that neutrophils are essential for innate defense against the protozoan parasite as GR-1 treated mice showed significant decreased cytokine expression, diminished T cell recruitment, and increased parasite burden.^12^^,^^13^ However, these interpretations were skewed due to this initial use of the GR-1 antibody, which binds not only the neutrophil-specific antigen, Ly6G, but also additional Ly6 family members, including Ly6C, a classic monocyte marker.^14^ To distinguish the individual role of neutrophils and monocytes during acute *T. gondii* infection, further studies were conducted by depleting neutrophils with the monoclonal antibody 1A8, which is specific to Ly6G.^15^ Neutrophil depletion using 1A8 showed nearly complete loss of Ly6G^+^ neutrophils while maintaining Ly6C^Hi^ monocytes and were able to control parasite replication with no change in host susceptibility.^15^ Meanwhile, mice administered GR-1 showed depletion of both Ly6G^+^ neutrophils and Ly6C^Hi^ monocytes, with a significant increase in parasite burden and rapid host mortality, suggesting that while neutrophils play a limited role in host defense, monocytes are an indispensable cell type for host resistance against *T*. *gondii.*^15^ Utilizing *Ccr2*^−/−^ mice, which demonstrated a lack of monocyte recruitment with no neutrophil defect, resulted in uncontrolled parasite replication and rapid host mortality, establishing that neutrophils play a limited role in host resistance against *T*. *gondii* infection.^15^

More recent studies have shown that neutrophils are capable of expressing IFN-γ during their development under homeostatic conditions.^16^ It has also been established that neutrophil-derived IFN-γ is TLR- and IL-12-independent; yet, IFN-γ secretion by neutrophils requires microbial or inflammatory environment-mediated degranulation of primary granules containing IFN-γ.^16^ Host defense against *T*. *gondii* is dependent on the TLR and IL-1R adaptor molecule MyD88, which is required for inducing IFN-γ in NK and T cells.^10^^,^^17^^,^^18^ Lack of MyD88 or IFN-γ results in rapid host mortality; however, in the absence of TLR11, the sensor for profilin recognition, the host remains resistant to *T*. *gondii* infection.^19^^,^^20^ Meanwhile, in the human genome, *TLR11* is a pseudogene and nonfunctional.^21^ Strikingly, depletion of NK cells or T cells from *Tlr11*^−/−^ mice did not affect overall IFN-γ production.^16^ Utilizing lymphoid-deficient (*Rag2*^−/−^*γc*^−/−^) mice it was identified that neutrophils are a critical source of IFN-γ during *T*. *gondii* infection and in the absence of TLR11 neutrophil-derived IFN-γ is essential for host resistance against parasitic infection.^22^ These results provide a potential mechanism for the human immune response against the parasite, wherein TLR11 is nonfunctional, as it is demonstrated that in the absence of TLR11-dependent IL-12 lymphocyte activation, neutrophil-derived IFN-γ is essential for host immunity during acute *T. gondii* infection.^22^

As previously mentioned, humans lack a functional version of TLR11 and completely lack TLR12 from their genome, suggesting that neutrophils could play an expanded role in human resistance against the parasite.^21^ During acute infection with *T. gondii*, human neutrophils are activated upon exposure to tachyzoites in vitro, leading to an increased expression of cell surface markers. Such markers include CD66b, CD11b, CD15, CD62L, and CD88, along with increased reactive oxygen species (ROS) production and decreased expression of CD62L, all indicative of neutrophil activation.^23^ NET release also occurred after active tachyzoite coculture with human neutrophils, confirming that *T*. *gondii* mediates the release of neutrophil-derived NETs.^23^ It was further demonstrated that supplementing human neutrophils with IL-12 was sufficient to induce IFN-γ production, building on in vivo studies conducted utilizing *Nocardia asteroides* and *Salmonella enterica* serovar Typhimurium (*S*. Typhimurium).^24–26^ Additionally, human neutrophil–derived IFN-γ could be further augmented with the addition of IL-2 and IL-15, indicating that neutrophil-derived IFN-γ in humans may play a more significant role in immunity against *T. gondii* than their TLR11-sufficient murine counterparts.^24^ While neutrophil-mediated host defense against *T*. *gondii* plays a limited role in murine survival, studies on the role of neutrophils in humans could open new avenues for initiating protective responses against this global protozoan.

## Monocytes

Monocytes are critical sentinels of the innate immune system that provide a rapid response to microbial infections. Following the immediate infection with *T. gondii*, monocytes are produced in the bone marrow via IL-6 signaling.^27^ Emergency myelopoiesis is stimulated, and through priming via peripheral NK cell–derived IFN-γ, monocytes egress from the bone marrow and are subsequently recruited to the site of infection.^28^^,^^29^ Traditionally, monocytes are classically thought to be precursors to both macrophages and DCs, termed monocyte-derived macrophages and DCs; however, murine studies have shown that these cell types differentiate independently of monocytes, while monocytes develop into their own dynamic phagocyte-related cells.^30–32^ These monocytic derivations contribute to various physiological processes in ways that mimic both macrophage and DC activity.

In mice, there are two primary subsets of monocytes: classical (inflammatory) Ly6C^Hi^ CCR2^Hi^CX3CR1^Lo^ and patrolling Ly6C^Lo^CCR2^Lo^CX3CR1^Hi^. Inflammatory Ly6C^Hi^ monocytes are well characterized, known to be rapidly recruited to the site of infection, and critical for host defense against *T*. *gondii*. In the setting of an acute mucosal *T. gondii* infection, uncontrolled expansion of Gram-negative bacteria of the Enterobacteriaceae family mediates significant intestinal immunopathology,^33–36^ and the resulting host response leads to rapid recruitment of neutrophils in order to limit commensal interaction with the intestinal epithelium,^35^ which is regulated by Ly6C^Hi^ inflammatory monocytes in a prostaglandin E_2_–dependent manner.^37^ In addition to being key regulators of neutrophil-mediated pathology during *T. gondii* infection, Ly6C^hi^ monocytes are also critical for host resistance due to their antiparasitic effector mechanisms, including IL-12, IFN-β, and TNF production; phagocytosis; and upregulation of inducible nitric oxide synthase (iNOS).^15^^,^^29^^,^^38^ iNOS generates NO a toxic metabolite that deprives *T. gondii*, an arginine auxotroph, of arginine, rendering the pathogen incapable of undergoing replication.^39–41^ While it has been shown that iNOS plays a limited role during acute infection, additional studies show that iNOS production is important for host defense during the chronic stage of infection, where mice succumbed to infection and possessed a much higher parasite burden.^42^ NO-dependent mechanisms have been indicated to be critical for host defense orchestrated by microglia in the CNS, as demonstrated by the presence of *T. gondii* in microglia treated with an NOS inhibitor.^42^^,^^43^

As Ly6C^Hi^ monocytes maintain host defense against *T. gondii*, they are also precursors to patrolling Ly6C^Lo^ monocytes. The comparatively longer-lived Ly6C^Lo^ monocytes reside in tissues to ensure epithelial integrity and utilize a “rolling” motion to patrol vasculature and rapidly respond to infection.^44^ Patrolling monocytes have been found to be continually present within the vasculature and can rapidly extravasate upon recognition of inflammatory chemokines. Ly6C^Lo^ patrolling monocytes are key in anti-inflammatory and healing processes. They inhibit the inflammatory response via anti-inflammatory mechanisms such as engulfing apoptotic T cells and inhibiting T cell function through anti-inflammatory factors and high-mobility group box 1 (CD52-HMGB1) binding.^45^ During the phagocytic process, Ly6C^Lo^ cells produce matrix metalloproteinases (MMPs), such as MMP9, MMP12, and MMP13, accelerating extracellular matrix degradation and inhibiting fibrosis.^45^ During tissue repair and reconstruction, Ly6C^Lo^ cells secrete hepatocyte growth factor and insulin-like growth factor to promote wound healing and tissue regeneration.^45^ Furthermore, it has been shown that patrolling monocytes play a critical role in preventing lung tumor metastasis via recruiting and activating NK cells.^46^ Additional studies are needed to define the wide breadth of patrolling monocytes' functionality to better understand their role in the innate immune system for microbial host defense.

Recent results have revealed that during *T. gondii* infection, there is a significant increase of Ly6C^Lo^ monocytes in the blood, CNS, and tissues that had yet to be infected, suggesting that patrolling monocytes play an important role in protecting host tissues during acute toxoplasmosis.^47^ Additional studies have shown that the *T. gondii–*derived protein kinase ROP17 can contribute to the migration of monocytes to sites of infection via ROCK (Rho-associated kinase) signaling, indicating a parasite-dependent and independent mechanism to the recruitment and migration of monocytes to different sites of infection.^48^  *E2^−/−^* or Nur77 knockout mice, which lack patrolling monocytes, can help us understand how patrolling monocytes defend against *T*. *gondii* in future studies.^46^^,^^49^

An important contribution to host protection is the monocyte’s ability to develop trained immunity. This form of immunity is hypothesized to involve activation via plasma-soluble transfer factors or cytokines such as IL-1 or TNF.^50^ While the mechanistic qualities of trained immunity remain unclear, a possible route could involve reprogramming the transcriptional profile of the cell in a manner similar to TLR-induced chromatin modifications.^51^ In vivo studies have illustrated that cellular promoters exhibit transcription factor recruitment, increased histone acetylation, H3K4 trimethylation, and chromatin remodeling upon initial exposure to lipopolyaccharide.^51^ The high H3K4 trimethylation with increased levels of histone acetylation and accessibility upon secondary exposure leads to faster kinetics and a more efficient innate immune response downstream of accelerated transcription of antimicrobial genes.^51^ Bridging the gap between innate and adaptive immunity, this process of trained immunity involves a more effective second-exposure response to pathogens without the somatic diversification present in the adaptive immune cells. An early study suggested the effect of “trained immunity” against *T. gondii* using CBA mice treated with muramyl dipeptide (MDP).^52^ MDP is recognized by NOD2 signaling through nuclear factor κB (NF-κB), which stimulates epigenetic rewiring of macrophages and induces trained immunity.^53^^,^^54^ This study shows that independently of enhanced anti-*Toxoplasma* antibodies, treating mice with MDP prior to infection with *T. gondii* lowered mortality rates,^52^ suggesting that another form of developed immunity to the pathogen, with trained immunity as a possibility.^50^ Further studies with soluble tachyzoite antigen (STAg) or *T*. *gondii*–derived profilin could prove to be effective in determining the nature of monocytic trained immunity against *T. gondii*.

Inflammasome recognition of intracellular pathogens is essential for innate defense as we have shown with *S*. Typhimurium and *T. gondii.*^20^^,^^55^ The inflammasome working in tandem with TLRs provides another aspect of monocyte-mediated host protection. Through the NLRP1 and NLRP3 inflammasomes, the adaptor molecule apoptosis-associated speck-like protein containing a CARD (ASC) is recruited that is essential for caspase-1 and caspase-11 activation, which then converts pro-IL-1β and pro-IL-18 into their biologically active forms.^20^^,^^56^^,^^57^ While the inflammasome has been established as indispensable for host defense against *S*. Typhimurium, our group and others have demonstrated that individual inflammasome components, NLRP3, ASC, and caspase-1 and caspase-11 play a limited role in host resistance during *T. gondii* infection.^20^^,^^58^^,^^59^ TLR11/12 recognition of *T*. *gondii* profilin initiates a robust MyD88-dependent, DC-derived, IL-12–mediated immunity against the parasite. However, in the absence of TLR11, mice do not demonstrate acute susceptibility, suggesting another innate protective pathway being concealed by a robust TLR/IL-12 response. Our group illustrates that in the absence of TLR11, it is IL-18 and not IL-1β that plays a critical role for Th1-mediated immunity during *T*. *gondii* infection.^20^ Additionally, a recent study demonstrates that it is the caspase-1–dependent release of IL-18 from Cx3cr1^+^ myeloid cells that plays a critical role in mediating Th1 effector function during *T*. *gondii* infection.^59^ Monocytes, macrophages, DCs, and microglia all express Cx3cr1 and have all been shown to play a vital role for myeloid cell–mediated defense against *T*. *gondii*, hence additional studies are needed to clarify the precise subpopulation of Cx3cr1^+^ cells, which are critical for innate inflammasome-dependent immunity.

It is important to note the suggested greater importance of inflammasome functionality in the context of human disease. During human *T. gondii* infection, IL-1β, a critical regulator of inflammation, is produced and secreted by monocytes in greater frequencies than IL-18, suggesting IL-1β having great impact on human monocyte-mediated immunity against *T. gondii.*^60^^,^^61^ Recent findings from the Yarovinsky group show that caspase-1 in human monocytes mediates the release of S100A11 from infected cells via the RAGE signaling pathway, triggering CCL2 production.^62^ These results highlight that monocyte infection by *T*. *gondii* is essential for a caspase-1–dependent, CCL2-mediated host defense mechanism. Utilizing S100a11-deficient mice, it was established that in vivo S100a11 promotes CCL2-mediated monocyte recruitment to the site of infection and significantly contributes to host survival.^62^

Studies investigating the *T. gondii* ligand recognized by the inflammasome have shown that neither heat-killed *T. gondii* nor mycalolide B–treated, invasion-inhibited parasites can induce IL-1β release,^20^^,^^60^ indicating that active cellular invasion is necessary for inflammasome activation. It was identified that in bone marrow–derived macrophages (BMDMs) from Lewis rats, NLRP1-dependent pyroptosis is mediated by direct infection and the parasite-dense granule proteins GRA35, GRA42, and GRA43.^63^ In human THP-1 monocyte cells infected with *T. gondii*, the AIM2 inflammasome sensor has been shown to be activated by *T. gondii* DNA that has been liberated by guanylate-binding protein (GBP) activity.^64^^,^^65^ It has also been shown using primary mouse peritoneal macrophages and BMDMs that NLRP3 was activated by extracellular adenosine triphosphate (ATP) signaling released from parasite-infected cells via the P2X7 receptor.^66^ Additionally, the dense granule protein, GRA15, from type II strains of the parasite, plays an important role for IL-1β release in both human monocytes and mouse BMDMs.^60^^,^^67^ Thus, the inflammasome component of monocyte-derived host defense could provide further clarification on human host defense mechanisms. Monocytes play an indispensable role in host defense against *T*. *gondii* by controlling parasite replication and facilitating innate recognition, which is critical for recruiting additional immune cells. Additionally, there is a significant gap in understanding the role of monocytes in parasite clearance during human infection due to the rapid immune response and the absence of *T. gondii–*specific clinical symptoms. Therefore, as we continue to understand the role of monocytes using the toxoplasmosis mouse model, it is necessary to simultaneously define the effector functions of human monocytes during acute infection.

## Macrophages

Macrophages play a central role in *T*. *gondii* clearance via multiple mechanisms including innate pathogen recognition, production of proinflammatory cytokines, NO, ROS, and induction of IFN-γ–mediated antiparasitic pathways. Early studies indicated that macrophages were a critical source of IL-12 during *T*. *gondii* infection,^68^ which mediated ILC-derived IFN-γ production. Murine studies have now established that the primary source of IL-12 during acute toxoplasmosis infection is produced by type I conventional DCs (cDC1s) through *T. gondii* profilin–activated TLRs 11/12.^17^^,^^19^^,^^69^ Further reports have linked additional *T*. *gondii*–derived PAMPs to inflammatory cytokine production by macrophages. *T*. *gondii*’s GPI and heat shock proteins have been shown to be recognized by TLR2 and TLR4 in macrophages, resulting in TNF and IL-12 production.^70–72^ Yet, studies demonstrate that TLR2 and TLR4 play a limited role in host defense, as *Tlr2*^−/−^ and *Tlr4*^−/−^ mice have no defect in CD4^+^ T cell–derived IFN-γ or effect on host survival.^70^^,^^71^ Additionally, it has been observed, using immortalized macrophages, that macrophages are capable of sensing *T*. *gondii* RNA via TLR7.^73^ Similar to TLR2 and TLR4 deficiency, lack of TLR7 does not confer susceptibility to *T*. *gondii* infection, nor does it result in an increase of tissue cysts.^73^ In the mouse model of toxoplasmosis, TLRs 2, 4, 7, and 9 appear to play a limited role in host resistance against *T*. *gondii* when TLR11 and TLR12 are intact. However, in the absence of TLR11, TLRs 3, 7, and 9 become essential for host defense during acute infection,^69^ indicating that in the absence of TLR11, non–profilin-recognizing TLRs play an important role for innate immunity toward *T*. *gondii*.

In addition to TLRs, macrophages are also capable of sensing *T. gondii* via the inflammasome. As previously stated, *T*. *gondii* can be recognized by both the NLRP1 and NLRP3 inflammasomes, mediating IL-1β and IL-18 production, in vitro and in vivo, respectively.^67^^,^^74^ In vivo studies of inflammasome-mediated protection have contradicting results, some studies indicate that the inflammasome is critical for limiting parasite replication and host survival,^67^ and others, including our group, have shown that the inflammasome plays a limited role in mediating CD4^+^ T cell–derived IFN-γ response and host resistance.^20^^,^^58^ Nevertheless, our previous findings identify that in the absence of TLR11, the inflammasome plays a significant role in mediating a robust Th1 response, most likely due to the significant upregulation of IL-18 observed in *Tlr11*^−/−^ mice during acute infection.^20^ It has also been reported that caspase-8 plays an important role in controlling the transcription of *Il12b* and *Il1b*, and mice lacking caspase-8 rapidly succumb to parasitic infection.^75^ Another reported mechanism of inflammasome-mediated host defense is the purinergic receptor P2X7R, an ATP-gated plasma membrane ion channel known to play a role in a wide range of host defense processes.^76^ P2X7R leads to the activation of the NLRP3 inflammasome and IL-1β secretion.^76^ Moreover, it was demonstrated, using macrophages, that in the absence of P2X7R, ATP-mediated parasite killing is significantly diminished.^77^

Early experiments utilizing human macrophages revealed that IFN-γ–mediated toxoplasmacidal activity is critical for the clearance of intracellular parasites.^78^ IFN-γ–primed murine macrophages are classically considered proinflammatory M1 macrophages capable of limiting *T*. *gondii* replication within the parasitophorous vacuole (PV).^79^ Furthermore, it was demonstrated that macrophages isolated from AIDS patients, when stimulated with IFN-γ, could eliminate intracellular *T*. *gondii*, demonstrating that these individuals had functional macrophages that are able to eliminate the parasite.^80^ Hence, IFN-γ–mediated macrophage-dependent cellular immunity plays a significant role in host resistance against *T*. *gondii* infection, with 5 major mechanisms triggered by IFN-γ. These IFN-γ induced mechanisms include (i) iNOS expression, depleting arginine via NO synthesis, starving *T*. *gondii*; (ii) ROS intermediates, which have been demonstrated to eliminate intracellular *T*. *gondii* in both IFN-γ–dependent and IFN-γ–independent pathways; (iii) inducible immunity-related GTPases (IRGs); (iv) p65 GBPs; and (v) IFN-γ–dependent and tissue-resident macrophage (TRM) cell death mediated via CD115 and the mTOR pathway. Similar to monocytes, macrophages utilize IFN-γ to induce expression of iNOS, depleting arginine via NO synthesis, starving *T. gondii.*^39^^,^^81^ ROS intermediates have also been demonstrated to eliminate intracellular *T*. *gondii* in both IFN-γ–dependent and IFN-γ–independent pathways.^82–84^ Thus, reasoning that in the absence of robust IFN-γ production, macrophages may remain capable of eliminating intracellular *T*. *gondii* via ROS production.

Another critical set of IFN-γ–inducible proteins are IRGs and p65 GBPs. IRGs including IRGM1 (also known as LRG47),^85^ IRGM3 (also known as IGTP),^86^^,^^87^ IRGD (also known as IRG47),^85^ IRGA6 (also known as IIGP1),^88^ and IRGB6 (also known as TGTP)^89^^,^^90^ all contribute to host-mediated defense and parasite clearance. GBPs are now understood to play a critical role in the recruitment of IRGs to the PV,^91^^,^^92^ leading to vesiculation of the PV membrane and, ultimately, parasite elimination. Moreover, it has been demonstrated that the autophagy protein ATG5 is required for IFN-γ–inducible IRGA6 recruitment to the PV membrane, mediating parasite clearance in the macrophage.^93^ Similarly, we have also demonstrated that ATG5 expression within Paneth cells is indispensable for tissue homeostasis in response to *T*. *gondii* infection, and the lack of ATG5 in either phagocytes or Paneth cells results in rapid host susceptibility.^93^^,^^94^ A limitation of the translational significance of IRG-related studies in mouse models is that 23 IRG genes are encoded in the mouse, whereas humans only retain one full-length IRG.^95^ Meanwhile, mice and humans possess 11 and 7 GBPs, respectively.^92^^,^^96^^,^^97^ Furthermore, it has been shown that murine GBP2 (mGBP2) targets the PV and promotes the recruitment of mGBP7 to elicit their antiparasitic function.^98^ It is important to note that the human orthologue of mGBP2, human GBP1 (hGBP1), has also been observed promoting the recruitment of hGBP2, hGBP3, hGBP4, and hGBP6 to *Shigella* in HeLa cells, demonstrating that GBP’s antimicrobial functions are conserved between different species.^99^^,^^100^

More recently, the role of TRMs has been explored in the context of *T*. *gondii* infection. These data suggest that the parasite-mediated immune response leads to TRM elimination in the peritoneal cavity, liver, and intestines during acute parasitic infection.^6^ TRM elimination was shown to be IFN-γ dependent and TRM cell death mediated via CD115 and the mTOR pathway, which appears to be a critical to remove a replicative niche of the parasite.^6^^,^^101^ Together, these data illustrate the multiple pathways that macrophages may utilize in actively eliminating the microbial niche and killing intracellular pathogens.

## Dendritic cells

As the classical antigen-presenting cells, DCs are drivers of the formation of the adaptive immune response. However, their primary role in innate immunity against *T*. *gondii* is initiating the IFN-γ–dependent immune response against the parasite. Early murine studies demonstrated that the adaptor molecule MyD88, which is downstream of most TLRs and IL-1 receptors, is critical for IL-12 production during *T*. *gondii* infection.^10^ It was then established using the mouse model of toxoplasmosis, *T*. *gondii* profilin is recognized by DCs via TLR11 and TLR12, mediating downstream MyD88- and UNC93B1-dependent signaling, critical for robust IL-12 production and IL-12–dependent Th1 effector function.^19^^,^^102^^,^^103^ It was then confirmed that TLR11 and TLR12 can both directly bind to profilin to form a heterodimer complex that also requires the presence of transcription factor IFN regulatory factor 8 (IRF8) for IL-12 production, rather than NF-κB signaling cascade.^103^ It is important to keep in mind, that unlike in mice, humans lack a functional TLR11 and completely lack TLR12 from their genome^21^; yet, humans that become infected with *T*. *gondii* are relatively resistant and typically asymptomatic to *T*. *gondii* infection, unless patients become immunocompromised. Recently, it was also shown that the transcription factor IRF5 also plays a role in IL-12 production by DCs and host resistance.^104^ However, it remains unclear if IRF5 is critical for all endosomal TLR recognition of *T*. *gondii* or is limited to TLR7 and TLR9, which has been observed in plasmacytoid DCs.^105^ Additional studies will be needed to dissect the role of IRF5 and IRF8 in cDC1-derived IL-12 production and host-mediated resistance.

Even though multiple myeloid cell populations can recognize *T*. *gondii* via TLRs (as described previously), multiple TLRs must work in concert to recognize *T*. *gondii* and induce a robust IL-12–dependent host immune response. The current innate immune signaling paradigm shows that the lack of MyD88 signaling in DCs using selective deletion largely recapitulates *T*. *gondii* infection in whole body *Myd88*^−/−^ mice,^17^ establishing the significance of DC TLR-MyD88-dependent recognition of the parasite to mediate host defense. Shortly after the need for MyD88 expression in DCs was observed, it was shown in murine studies that CD8a^+^ DCs are the primary source of IL-12 following STAg administration.^106^ It was then confirmed by us and others that CD8a^+^Batf3^+^IRF8^+^ cDC1-derived IL-12 is critical to initiate a robust IL-12–dependent ILC1-, NK-, and Th1-derived IFN-γ response.^69^^,^^107^^,^^108^ Meanwhile, it has been demonstrated that a subset of KLF4-expressing cDC2s do not play a major role in controlling parasite replication or host survival.^109^ Overall, these in vivo studies demonstrate that IRF8^+^ cDC1s are critical in mediating host resistance against *T*. *gondii* and cDC2s play a lesser role in host defense against the parasite.

Recently, our group demonstrated that T-bet–dependent ILC1-derived IFN-γ, and to a lesser extent NK cell–derived IFN-γ, play a major role in maintaining inflammatory IRF8^+^ cDC1s during *T*. *gondii* mouse i.p. infection model and restricting parasite growth in peripheral tissues.^108^ Following this study, we investigated whether ILC1s were required for host immunity during *T*. *gondii* infection. Surprisingly, *Tbx21*^−/−^ mice, which lack ILC1s and retain NK, T, and B cells, succumbed significantly quicker to infection than *Rag2*^−/−^*γc*^−/−^ mice, that lack all lymphocytes, suggesting that T-bet–expressing myeloid cells play a role in parasite elimination and overall host defense.^4^ Our group recently demonstrated that IL-12 is required for mediating a subpopulation of TMCs. In the following section, we examine the known role of T-bet in mediating immunity against *T*. *gondii* and the precedence for a T-bet–expressing myeloid cell population to assist in host resistance against infectious disease.

## T-bet–mediated immunity against *T*. *gondii*

**Figure 2.**
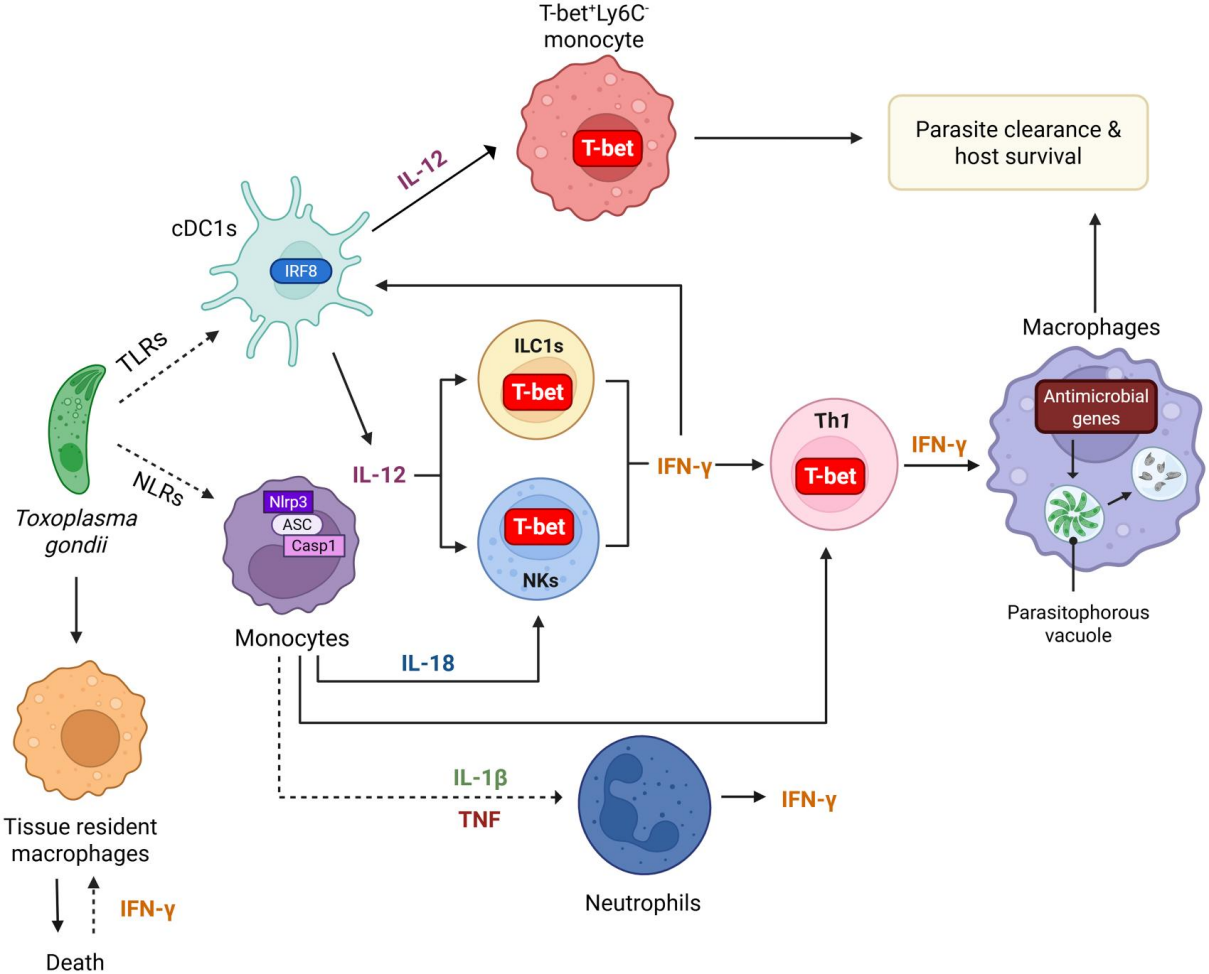
T-bet–mediated host defense during *T. gondii* infection. The transcription factor T-bet is critical for host survival. Here we illustrate the involvement of T-bet in the host immune response against *T. gondii*. Recognition of the parasite by myeloid cells, via TLRs and NLRs, contributes to a robust protective response, maintained through production and release of IL-12 and IL-18. The effector function of T-bet–dependent ILC1s and NK cells is dependent on myeloid cell–derived IL-12 and IL-18. T-bet orchestrates the innate immune response through expression in ILC1s, NK cells, Th1 cells, and a recently defined population of TMCs, that are mediated by IL-12. While additional innate immune cells are required for overall host survival, recent studies have demonstrated that mice conditionally lacking T-bet within CD11c^+^ cells succumb to infection at the same rate as *Tbx21*^−/−^ mice, suggesting that TMCs may play a crucial role in host defense. Upregulation of IFN-γ is another critical component of host defense; its classical production by ILC1s, NK cells, and Th1 cells. A novel finding of neutrophil-derived IFN-γ provides a method for production of this cytokine in a TLR- and IL-12–independent manner. IFN-γ also promotes TRM cell death, a protective measure aimed at limiting parasite replication within the phagocyte population. Created using BioRender (https://BioRender.com/x12o358).

*T*. *gondii* infection mediates a robust type I immune response defined by the effector functions of lymphocytes, including ILC1s, NK cells, and CD4^+^ Th1 cells.^102^^,^^110–116^ The transcription factor T-bet, encoded by *Tbx21*, is considered essential for the development and function of ILC1s, NK cells, and CD4^+^ Th1 cells.^110^^,^^117–119^ Classically, T-bet has been considered the master regulator of IFN-γ expression by CD4^+^ Th1 cells.^117^ However, our group and others have shown that T-bet is not required for parasite-mediated NK cell–, CD8^+^ T cell–, or Th1-derived IFN-γ, demonstrating that T-bet is largely dispensable for overall IFN-γ production^2^^,^^34^^,^^108^ [For an in-depth review of T-bet’s function in lymphocytes, see Harms Pritchard et al.^5^]. Our group was the first to demonstrate a role for T-bet in myeloid cell–mediated host defense against *T*. *gondii* (Fig. 2)^4^; however, identifying T-bet expression in myeloid cells is not uncommon. Preceding reports have demonstrated that T-bet expression in DCs may be upregulated in response to IFN-γ.^120^ Moreover, T-bet expression in DCs has been shown to play a role in controlling inflammatory arthritis, priming of antigen-specific T cells, CpG DNA adjuvancy, and IFN-γ production.^121–123^ Seminal studies examining the role of T-bet in innate immunity in the absence of T and B cells led to significant findings demonstrating that T-bet expression within DCs is critical to limit their TNF production.^124^^,^^125^ Moreover, in the absence of T-bet–expressing DCs, as well as T and B cells, the host will develop spontaneous communicable ulcerative colitis that will progress to colonic dysplasia and rectal adenocarcinoma due to MyD88-independent intestinal inflammation.^124^^,^^125^ Moreover, if the host’s DCs are engineered to overexpress T-bet in the absence of T and B cells, these mice displayed a significant reduction of neoplasia. In parallel to the role T-bet plays in restricting DC-derived TNF, recent results have identified two major cDC2 subsets that differentially express T-bet and RORγt, cDC2A, and cDC2B, respectively.^126^ In agreement with previous reports, T-bet–expressing cDC2As are anti-inflammatory and RORγt^+^ cDC2Bs are proinflammatory, expressing TNF and suggesting that in the absence of T-bet cDC2s will default to TNF expressing RORγt^+^ cDC2Bs exacerbating colonic inflammation.^126^ Additional studies are needed to confirm if *Tbx21*^−/−^ mice only have cDC2Bs.

**Figure 3.**
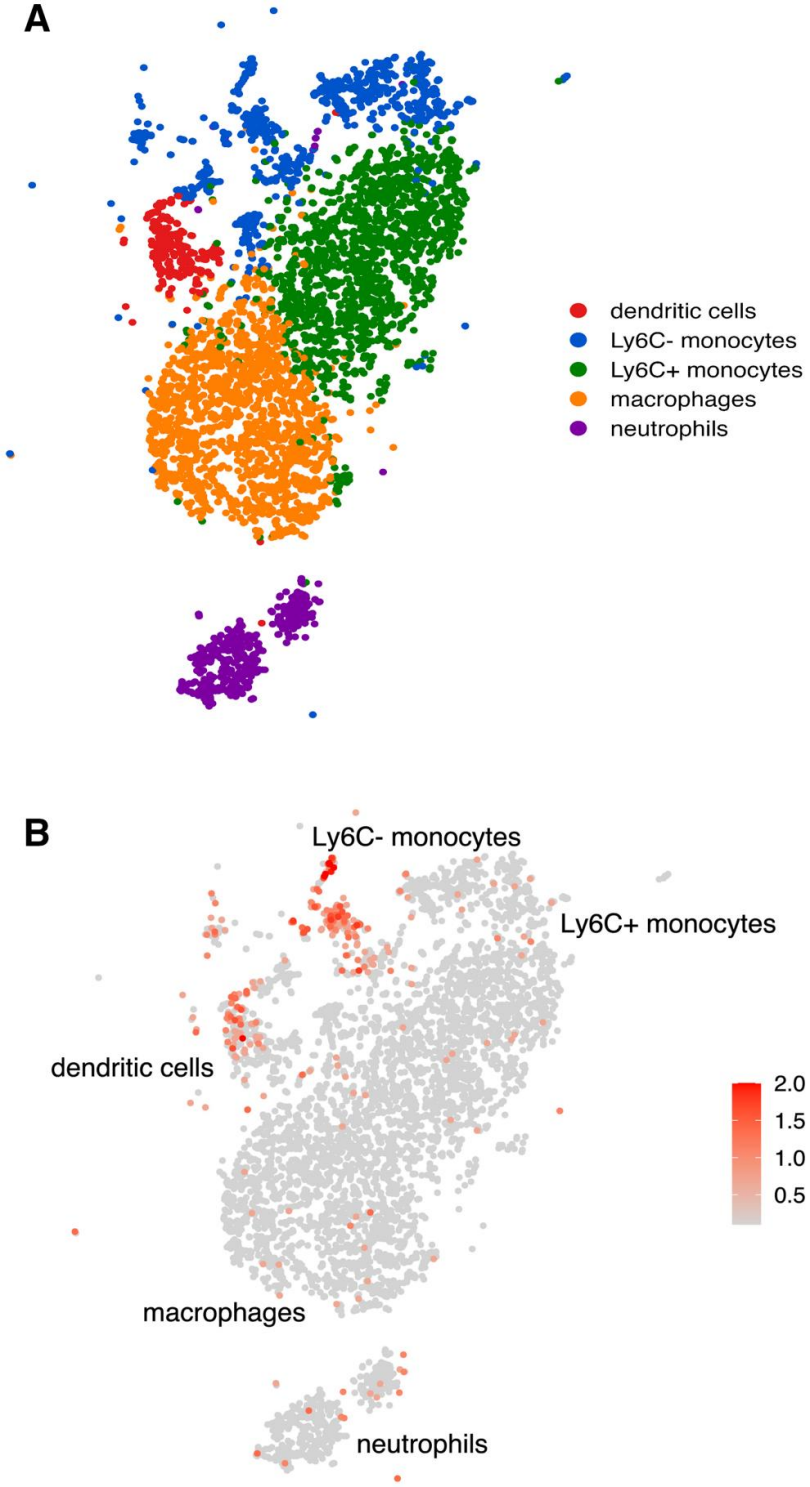
Frequency of T-bet expression within myeloid populations. C57BL/6J mice were i.p. infected with 20 cysts and peritoneal exudate cells were harvested on day 5 post-infection. CITE-seq was performed by using TotalSeq antibody cocktail (BioLegend) according to the manufacturer’s instructions. Raw FASTQ files were mapped to the GRCm39 reference genome using 10x Genomics Cell Ranger 9.0.0^134^ to infer read counts of both gene expression (RNA) and antibody-derived tag per gene per cell. Quality control is performed using Seurat package v5.0^135^ by removing cells with fewer than 500 or more than 7,500 detected genes, and cells fewer than 1,000 or more than 50,000 unique molecular identifiers, and cells greater than 20% mitochondrial content. Following quality control, SCTransform^136^ was used for normalizing the RNA read counts. Principal component analysis (PCA) is performed for dimension reduction on the normalized RNA read counts for the purpose of cell clustering. Specifically, the top 30 principal components were used with a resolution of 0.5. (A) t-Distributed stochastic neighbor embedding for cluster visualization. Antibody-derived tag counts were normalized using the centered log ratio method. Clusters were further annotated based on known marker genes or antibody levels. (B) Particularly, *Tbx21* expression levels were subsequently visualized across annotated clusters. Through unpublished CITE-seq results, our group has identified a strong expression of *Tbx21* within a subset of Ly6C^Lo-neg^ monocytes and DCs. While limited expression exists within other cell types, a majority of T-bet in myeloid populations is found within a distribution that we believe contains TMCs. Further analysis is being conducted to determine the qualities of this population and isolate them for further experiments to determine their nature of functionality.

An unpublished observation from the Glimcher group^125^ and our own recent results indicate that not only macrophages, but also Ly6G^+^ neutrophils, Ly6C^Hi^ monocytes, and DCs have limited T-bet expression. Unexpectedly, we observed a unique subpopulation of T-bet expressing CD45^+^CD3^−^CD19^−^Nkp46^−^F4/80^−^Ly6C^Lo^Ly6G^−^CD11c^+^MHCII^−^ myeloid cells, which we have termed TMCs, which had the largest intracellular parasite burden within the peritoneum following an i.p. infection relative to other professional phagocytes, suggesting they are critical for *T*. *gondii* elimination. Additionally, we have observed TMCs in the small intestinal lamina propria and spleens of infected mice following a mucosal infection.^4^ Further studies are needed to determine if TMCs in the lamina propria also have the highest frequency of parasite burden during acute *T*. *gondii* infection. To better delineate if these TMCs are a subpopulation of an established myeloid cell, we have performed cellular indexing of transcriptomes and epitopes (CITE-Seq) from peritoneal leukocytes on day 5 of i.p. *T*. *gondii* infection. From this, we observed that both DCs and Ly6C^Lo-neg^ monocytes have robust T-bet expression compared with neutrophils, Ly6C^Hi^ monocytes, and macrophages (Fig. 3). These observations indicate that during *T*. *gondii* infection there are two primary populations of T-bet–expressing myeloid cells, DCs, and Ly6C^Lo-neg^ monocytes. Following these results, the indicated populations are potentially (i) downregulating major histocompatibility complex class II (MHCII), (ii) downregulating Ly6C and upregulating CD11c, or (iii) a novel subpopulation of circulating T-bet–expressing Ly6C ^Lo-neg^ patrolling monocytes. In the following section, we examine each of these possibilities and propose potential experiments to test them. (i) As we have discussed, there is extensive literature that DCs, specifically cDC2As, express T-bet, and in the absence of T-bet the host displays uncontrolled DC-derived TNF production that can ultimately result in neoplasia.^124^^,^^125^ Additionally, it has been demonstrated that antigen-presenting cells (APCs) infected with *T*. *gondii* will lead to the downregulation of MHCII, possibly leading to the T-bet^+^CD11c^+^MHCII^−^ population we have observed.^127^^,^^128^ Yet, this is unlikely based on our results using T-bet reporter mice, which indicate there is no gradient of either MHCII or T-bet expression during infection. To determine if TMCs are derived from MHCII^+^ APCs a bone marrow (BM) chimera model could be utilized. As antigen presentation is critical to develop a robust immune response, we will transfer BM from LysM- and CD11c-Cre mice crossed with *MHCII*^fl/fl^ to conditionally delete MHCII from neutrophils, monocytes, and macrophages (M-*MHCII*^−/−^), or DCs and TMCs (CD11c-*MHCII*^−/−^). We will use B6 CD45.1 as recipients and transfer BMs of M-*MHCII*^−/−^ + B6 CD45.2 (M + B.2→B.1) or CD11c-*MHCII*^−/−^ + B6 CD45.2 (CD11c+B.2→B.1). Eight weeks following the transplantation, we will infect M + B.2→B.1, CD11c+B.2→B.1, M-*MHCII*^−/−^, CD11c-*MHCII*^−/−^, and B6 CD45.1 with 20 cysts i.p. and by flow cytometry on day 5 postinfection assess the presence of TMCs from the peritoneum. We anticipate that we will not observe any defect in the frequency or total cell number of TMCs at day 5 postinfection from the infected transplant groups or the B6 controls. Additionally, we expect that conditionally deleting MHCII expression from APCs using either M-*MHCII*^−/−^ or CD11c-*MHCII*^−/−^ mice will result in a significant defect in the Th1 effector response, resulting in an increase in pathogen burden, and as a result will display significantly attenuated frequency and absolute cell numbers of TMCs during infection. (ii) Studies have demonstrated that during *Leishmania* and *T*. *gondii* infection, Ly6C^+^ monocytes downregulate Ly6C and upregulate CD11c.^129^^,^^130^ During *T*. *gondii* infection, myeloid cells were able to be differentiated as Ly6C^int^CD11c^+^MHCI^+^MHCII^+^ and Ly6C^−^CD11c^+^F4/80^+^TREM2^+^, suggesting they take on DC-like characteristics and have elevated phagocytic capability, respectively.^129^ Hence, the previously described Ly6C^−^CD11c^+^F4/80^+^TREM2^+^ monocytes could be the TMCs that we have observed, but based on our gating strategy that excludes F4/80^+^ cells, it is doubtful that these are the same population. To determine if TMCs differentiate from Ly6C^+^ monocytes, we have generated LysM-Cre x *Tbx21*^fl/fl^ (M-*Tbx21*^−/−^) mice, conditionally deleting T-bet expression from neutrophils, monocytes, and macrophages. Our previous findings demonstrate that conditional deletion of T-bet in neutrophils, monocytes, and macrophages did not lead to increased parasite burden or accelerated host mortality during infection.^4^ However, conditionally deleting T-bet from CD11c^+^ cells resulted in a significant increase of parasite burden at the site of infection compared with T-bet–sufficient mice.^4^ We have also observed (unpublished observation) that infected M-*Tbx21*^−/−^ mice displayed no defect in the frequency or absolute cell numbers of TMCs compared with wild-type controls on day 5 postinfection. These preliminary observations suggest that TMCs are not differentiated from Ly6C^+^. As Ly6C^Lo^ monocytes are not solely derived from downregulating Ly6C,^131^ these results suggest another pathway for TMC development via the common monocyte progenitor (cMoP). (iii) Patrolling Ly6C^Lo^CCR2^Lo^Cx3cr1^Hi^ monocytes have been described as the myeloid population that “patrol” the host’s vessels during healthy conditions, and during tissue damage or infection they are capable of rapidly responding to the insult and differentiating into macrophages.^131^ Recently, it was observed that there is a significant increase of Ly6C^Lo^ monocytes in both the blood and the brain during acute *T*. *gondii* infection,^47^ and in our own studies, it appears that Ly6C^Lo^ monocytes, potentially TMCs, enter the peritoneum rapidly and play a critical role in host defense against *T*. *gondii*. Moreover, based on our CITE-seq observations (Fig. 3), we have found that Ly6C^Lo^ monocytes contribute to host defense against intracellular pathogens in a T-bet–dependent pathway. As previously mentioned, patrolling monocytes can be generated via 2 pathways: (i) through direct differentiation from a cMoP or (ii) further differentiation of Ly6C^+^ monocytes to Ly6C^Lo^.^132^ cMoPs are a subset of the monocyte-DC precursor, which are capable of producing both patrolling and inflammatory monocytes but do not contribute to DC production.^132^ Regardless of origin, patrolling monocytes have been shown to be dependent on the transcription factor Nur77 for development yet play no role for the development or function of inflammatory monocytes.^132^ Therefore, to determine whether patrolling monocytes are critical for host defense against *T. gondii,* it will be imperative to test the acute toxoplasmosis mouse model employing Nur77 knockout (*Nr4a1^−/−^*) mice.^46^^,^^49^ Based on our previous findings, we predict that i.p.-infected *Nr4a1^−/−^* mice would have a significant increase in parasite burden, nearly a complete loss of TMCs, and succumb rapidly to infection, similar to *Tbx21^−/−^* mice, suggesting that TMCs are a subset of patrolling monocytes and have a significant role for host resistance against *T. gondii.* To further determine if TMCs are a subpopulation of patrolling monocytes, adoptive transfer studies would be performed in which patrolling monocytes from B6 or *Tbx21^−/−^* mice are transferred to *T. gondii* infected *Nr4a1^−/−^* mice and their pathogen burden and frequency of TMCs quantified on day 5 post-infection. As patrolling monocytes have been demonstrated to be strategically located throughout the host prior to infection, a confounding variable with the adoptive transfers would be the lack of their natural seeding throughout the host. While the transferred patrolling monocytes may still contribute to parasite clearance, their response might be less robust systemically, as they were not initially distributed according to their natural pattern. Further analysis could involve utilizing a BM transplant, with irradiated *Nr4a1^−/−^* mice that would receive BM transfers from either wild-type or *Tbx21*^−/−^ donors. This approach to identify the lineage of TMCs could also be combined with the use of a Nur77-GFP reporter strain, which have the Nur77 promoter driving expression of a GFP indicating whether TMCs have expressed Nur77 at any point in their development; yet, although this would only provide a correlative observation between Nur77 expression and TMCs, it does provide further direction for classifying this cell type. Overall, these experiments would provide further direction for mechanistic studies of this population and give guidance for determining a similar population that may exist in humans.

## Conclusions and perspectives

Inspection of the commonalities that mediate immunity is critical for the development of a comprehensive model of a coordinated and protective immune response. One such commonality is the expression of T-bet within myeloid populations, which is highly conserved across species, including humans.^133^ Several studies have identified that T-bet can be expressed in DCs and monocytes, and plays a role in controlling inflammatory arthritis, priming of T cells, CpG DNA adjuvancy, IFN-γ and TNF production, colorectal cancer development, and host defense against *T*. *gondii*. Using CITE-seq, T-bet expression was identified in various myeloid populations during acute *T. gondii* infection including DCs and Ly6C^Lo-neg^ monocytes (Fig. 3). This observation generates novel questions in the field: (i) Does T-bet directly mediate cell-intrinsic antimicrobial defense for parasite clearance? (ii) Do T-bet–expressing myeloid cells augment the CD4^+^ T cell effector function during infection? and (iii) Do humans possess T-bet–expressing myeloid cells that can eliminate intracellular pathogens? Moreover, this new avenue of research has far-reaching translational implications for targeting T-bet–dependent myeloid cell immunity against *T*. *gondii*, as well as other highly virulent intracellular human pathogens, such as *Leishmania*, *Mycobacteria*, and *Salmonella*.

The recent identification of key effector mechanisms of neutrophils, monocytes, macrophages, and DCs against acute *T. gondii* infection may inform other long-standing questions about the role of myeloid cell populations in host resistance to intracellular pathogens. Our recent study has revealed T-bet to be vital for CD11c^+^MHCII^−^ myeloid cells in initiating and maintaining protective mechanisms against *T. gondii*. It is imperative to further define the importance of T-bet within the myeloid cell populations. Our study found TMCs do not only possess high levels of T-bet, but also the highest levels of parasite burden, suggesting that this population of cells is an unexplored and critical component of host defense against *T. gondii*. We continue to build on these observations by leveraging CITE-seq to further define the qualities of T-bet–expressing myeloid cells. Future studies will be aimed at delineating the overall contribution to host protection. While the human immune system lacks key components of the anti-*T. gondii* murine immune system, such as TLR11, they are still capable of mounting immune responses that clear and control intracellular parasites such as *T. gondii*. Thus, focused murine studies can be used to determine the full extent of TMCs’ effector function to extrapolate which processes are indispensable for defense against intracellular pathogens. Defining the mechanisms by which neutrophil, Ly6C^Hi^ monocyte, Ly6C^Lo^ monocyte, TRM, macrophage, and DC populations individually contribute to the innate immune response offers critical insight into developing novel parasitic prevention methods and therapies.

## Data Availability

The data underlying this article is included in this article.
